# Comparison of the prognostic value of impaired stress myocardial blood flow, myocardial flow reserve, and myocardial flow capacity on low-dose Rubidium-82 SiPM PET/CT

**DOI:** 10.1007/s12350-022-03155-6

**Published:** 2022-12-27

**Authors:** Matthieu Dietz, Christel H. Kamani, Gilles Allenbach, Vladimir Rubimbura, Stephane Fournier, Vincent Dunet, Giorgio Treglia, Marie Nicod Lalonde, Niklaus Schaefer, Eric Eeckhout, Olivier Muller, John O. Prior

**Affiliations:** 1 Nuclear Medicine and Molecular Imaging Department, Lausanne University Hospital, Rue du Bugnon 46, 1011 Lausanne, Switzerland; 2 INSERM U1060, CarMeN Laboratory, University of Lyon, Lyon, France; 3 Department of Cardiology, University Hospital of Lausanne, University of Lausanne, Lausanne, Switzerland; 4 Nuclear Medicine Department, Fribourg Hospital HFR, Fribourg, Switzerland; 5 University of Lausanne, Lausanne, Switzerland; 6 Department of Diagnostic and Interventional Radiology, Lausanne University Hospital, Lausanne, Switzerland; 7 Clinic of Nuclear Medicine, Imaging Institute of Southern Switzerland, Ente Ospedaliero Cantonale, Bellinzona, Switzerland; 8 Università Della Svizzera Italiana, Lugano, Switzerland

**Keywords:** Quantitative myocardial perfusion, myocardial flow capacity, low-dose rubidium-82, major adverse cardiovascular events, outcome, SiPM PET/CT

## Abstract

**Background:**

The most reliable quantitative variable on Rubidium-82 (^82^Rb) cardiac PET/CT for predicting major adverse cardiovascular events (MACE) has not been characterized with low-dose silicon photomultipliers (SiPM) technology, which allows halving injected activity and radiation dose delivering less than 1.0 mSv in a 70-kg individual.

**Methods and Results:**

We prospectively enrolled 234 consecutive participants with suspected myocardial ischemia. Participants underwent ^82^Rb cardiac SiPM PET/CT (5 MBq/kg) and were followed up for MACE over 652 days (interquartile range 559-751 days). For each participant, global stress myocardial blood flow (stress MBF), global myocardial flow reserve (MFR), and regional severely reduced myocardial flow capacity (MFC_severe_) were measured. The Youden index was used to select optimal thresholds.

In multivariate analysis after adjustments for clinical risk factors, reduced global stress MBF < 1.94 ml/min/g, reduced global MFR < 1.98, and regional MFC_severe_ > 3.2% of left ventricle emerged all as independent predictors of MACE (HR 4.5, 3.1, and 3.67, respectively, *p* < 0.001). However, only reduced global stress MBF remained an independent prognostic factor for MACE after adjusting for clinical risk factors and the combined use of global stress MBF, global MFR, and regional MFC_severe_ impairments (HR 2.81, *p* = 0.027).

**Conclusion:**

Using the latest SiPM PET technology with low-dose ^82^Rb halving the standard activity to deliver < 1 mSv for a 70-kg patient, impaired global stress MBF, global MFR, and regional MFC were powerful predictors of cardiovascular events, outperforming traditional cardiovascular risk factors. However, only reduced global stress MBF independently predicted MACE, being superior to global MFR and regional MFC impairments.

**Graphical Abstract:**

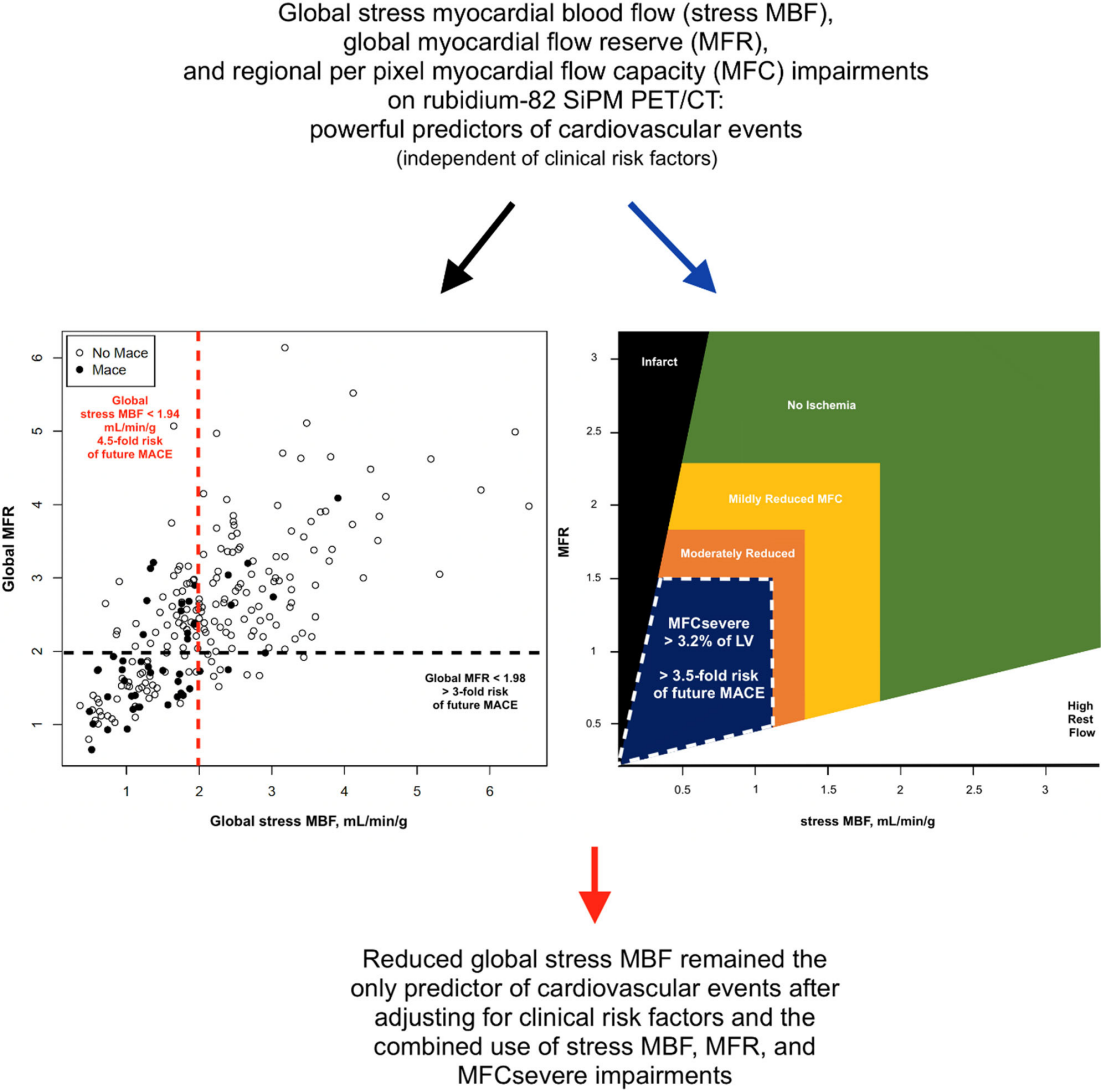

**Supplementary Information:**

The online version contains supplementary material available at 10.1007/s12350-022-03155-6.

## Introduction

Myocardial perfusion imaging is a powerful non-invasive functional tool for risk stratification, recommended by clinical practice guidelines.^1–5^ Compared with relative perfusion images, absolute quantification of myocardial blood flow (MBF) by positron emission tomography (PET) could improve risk stratification.^6^ Global and regional perfusion provide information on different aspects of myocardial perfusion. Impairment in global perfusion may be caused by either multivessel epicardial disease or microcirculatory dysfunction. Regional absolute perfusion measurements may enable the additive detection of small regional defects caused by epicardial coronary artery disease (CAD), which could not be detected with average global perfusion measurements.^6^

The myocardial flow capacity (MFC) concept is a precise regional approach integrating both MFR and stress MBF through the pathophysiologic severity of CAD to depict regional quantitative flow metrics on a per pixel basis.^6,7^ MFC may overcome some of the limitations of using stress MBF or MFR alone and represents a promising tool to improve clinical decision-making.^7–9^ However, despite a robust conceptual validation and recently promising clinical data especially after revascularization, further validations of the prognostic potential of MFC in comparison with stress MBF and MFR are still needed.^8–10^

Silicon photomultipliers with digital readout (SiPM) PET represents a major advancement in PET technology. This new system including smaller crystals exhibits a much higher sensitivity and outperforms previous PET scanners using conventional photomultiplier tubes according to essential PET parameters such as spatial and timing resolution or noise-equivalent count-rate.^11^ Moreover, this novel technology allows for the reduction of the standard dose, with an improved image quality.^12^ Although research about the diagnostic accuracy of this dedicated SiPM PET system has been performed in a preliminary comparative study with a small sample size,^13^ no previous study has evaluated the prognostic value of PET myocardial perfusion imaging with SiPM.

The aim of this study was to prospectively compare on a low-dose SiPM PET camera, halving the activity and radiation dose of Rubidium-82 (^82^Rb), the prognostic value for cardiovascular events of stress MBF, MFR, and MFC.

## Methods

### Study population

We prospectively enrolled participants with clinical suspicion of myocardial ischemia (at the discretion of the referring clinician) to undergo 82Rb cardiac SiPM PET/computed tomography (CT) between June 2018 and June 2019 at the Lausanne University Hospital. Participants’ cardiovascular risk factors and medication use were ascertained at time of PET imaging. A history of CAD (“known CAD”) was defined as evidence of myocardial infarction (MI), previous percutaneous coronary intervention (PCI) or coronary artery bypass graft (CABG), or angiographically significant coronary stenosis (> 50% of the left main coronary artery or > 70% stenosis in any epicardial coronary artery). All procedures performed in this study were in accordance with the 1964 Helsinki declaration and its last amendments or comparable ethical standards. The Local Ethics Committee approved this study protocol (#PB_2017-00,634), and all participants gave written informed consent prior to inclusion.

### Imaging protocol with SiPM ^82^Rb PET/CT

For each participant, a rest and adenosine or regadenoson stress SiPM PET/CT scan was performed, using a single dedicated camera (Biograph Vision 600, Siemens Medical Solutions, Knoxville, USA). Participants were instructed to fast for 6 h and avoid caffeine-containing food or beverages 24 h prior to the test. At rest, a 15-25 s intravenous (i.v.) infusion of low-dose (5 MBq/kg) ^82^Rb (Ruby-Fill® generator and ^82^Rb elution system [v3], Jubilant DraxImage, Kirkland, QC, Canada) was administered with an automatic infusion system and three-dimensional (3D) dynamic PET images were acquired starting at the beginning of the infusion over 6 min 19 s (12 × 8, 5 × 12, 1 × 30, 1 × 60, and 1 × 120 s). A second acquisition was then started following the same protocol with similar activity 2 min after the beginning of an adenosine infusion (140 mg/kg/min over 6 min) or following a regadenoson administration (400 µg over 10 s). A low-dose CT (100 keV, 16 mAs) transmission scan was used for attenuation correction. Images were reconstructed by ordered subsets expectation maximization algorithms (4 iterations, 5 subsets, 4.0 mm Full Width at Half Maximum (FWHM) Gaussian post-filter, 220 × 220-pixel matrix size). Blood pressure, heart rate, and a 12-lead ECG were recorded throughout the procedure. The radiation dose for a 70 kg participant was estimated to be 2 × 0.39 mSv for rest and stress ^82^Rb, and 1 × 0.17 mSv for the low-dose attenuation correction CT plus CT scout, resulting in a total dose of 0.95 mSv.

### Usual quantitative myocardial perfusion analysis

Perfusion was assessed quantitatively measuring MBF in milliliter per minute per gram at rest and stress, using the highly automated FlowQuant v2.7 software (Ottawa, Ontario, Canada), with a 1-tissue compartment model with a flow-dependent extraction correction.^14^ MFR was calculated as follows: MFR = stress MBF/rest MBF. Rate-pressure product-adjusted rest MBF and MFR were determined to account for high resting heart rate or systolic blood pressure by multiplying rest MBF by 8500 mmHg/min and dividing by rate-pressure product (resting heart rate multiplied by resting systolic blood pressure). To reduce the potential spill-over in image-derived blood activity curves, a dual spill-over correction was systematically applied.^15^ Global partial-volume recovery correction and motion correction were also systematically applied.^16^

### Myocardial flow capacity

MFC, developed by Johnson and Gould using ^82^Rb PET imaging, is a metric that integrates per pixel combination of resting MBF, stress MBF, and MFR into pathophysiologic severity categories by an integrated color map.^7–9^ MFC pixels (n = 513 on the polar map) having both MFR ≤ 1.5 and stress MBF ≤ 1.1 mL/min/g were defined as severely reduced MFC (MFC_severe_) and were quantified as percent of left ventricle (LV). Because regional MFC_severe_ was the MFC category previously associated with the higher risk among all MFC category, only regional MFC_severe_ was included for MFC in this prognostic study.^8,9^

### Clinical follow-up

The endpoint of the study was major adverse cardiovascular event (MACE), defined as cardiac death, MI, delayed revascularization (> 6 months post-PET/CT), hospitalization for congestive heart failure, or de novo stable angina. Early revascularizations observed within the first 6 months post-PET/CT were considered to have been triggered by the myocardial perfusion study and were excluded. Death from cardiac cause was defined as death from MI, congestive heart failure, valvular heart disease, sudden death, death without a witness or of unknown cause, and cardiac interventional/surgical procedure related. Hospitalization for de novo stable angina was defined as angina or chest pain of cardiac origin and requiring further investigations and hospitalization. Outcome information was obtained from medical records available in the hospital information system. If unsuccessful, participant follow-up was obtained by a phone call to cardiologists or general practitioners and/or participants. In participants with multiple MACE, only the first one was considered for survival analysis. Outcome data were collected from January to February 2021.

### Statistics

We assessed the distribution of data with the Shapiro-Wilk test. Continuous normally distributed variables were presented as mean ± SD and compared using Student’s t-tests. Continuous non-normally distributed variables were presented as median [interquartile range] and compared using the Mann-Whitney U test. The chi-square test or Fisher exact test was used for analysis of categorical variables.

The Youden index was used to select optimal thresholds based on receiver operating characteristic curves for stress MFR, MFR, and MFC_severe_ measurements. Kaplan-Meier curves were used to elucidate the survival distributions regarding MACE. Differences in the outcomes of participants were assessed using the log-rank test. A Cox proportional hazard regression with adjustment for potential confounders was performed to determine the predictors of worse outcome. To prevent overfitting of the multivariate Cox proportional hazards models, only cardiovascular risk factors with *p* values < 0.05 in univariate Cox proportional regression models were considered in the multivariate models.

Collinearity between global stress MBF, global MFR, and regional MFC_severe_ was assessed by calculating the variance inflation factors in the final model (lower than five for each variable).^17^

The statistical analysis was performed using R version 4.1.1 (R Foundation for Statistical Computing, Vienna, Austria). All *p* values used were two-sided, with *p* < 0.05 considered statistically significant.

## Results

### Participant’s characteristics

**Figure 1:**
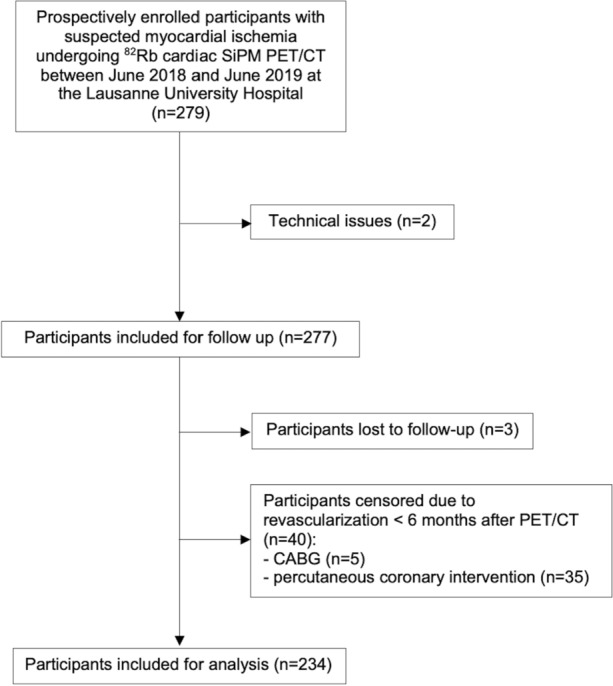
Study flowchart. CABG - coronary artery bypass graft; CT - computed tomography; PET - positron emission tomography; ^82^Rb - rubidium-82; SiPM - silicon photomultipliers with digital readout

The flowchart of the study is shown in Fig. 1. From June 2018 to June 2019, low-dose ^82^Rb SiPM PET/CT was performed in 279 participants. Two studies were excluded because of technical issues (delayed imaging after infusion start). Follow-up was successful in 274 of 277 remaining participants (99%). 40 participants were censored due to early revascularization (5 CABG surgery and 35 PCI, < 6 months after PET/CT). Baseline characteristics of the remaining study population of 234 participants are given in Table 1. Participants had a high prevalence of known CAD (54%), with a high burden of cardiovascular risk factors (hypertension: 73%; current or former tobacco use: 45%; dyslipidemia: 68%; diabetes: 36%). Preventive therapies were highly prescribed in the overall population: 58% with aspirin, 62% with beta-blockers, 58% with angiotensin-converting enzyme inhibitors/angiotensin receptor blockers, and 66% with lipid-lowering agents.

**Table 1:** Baseline clinical characteristics

	Overall population (n = 234)	MACE (n = 47)	No MACE (n = 187)	p-value
Age, years, median [IQR]	72 [61-78]	73 [68-79]	71 [60-77.5]	0.105
Male sex, n (%)	153 (65%)	36 (77%)	117 (63%)	0.081
Body mass index, kg/m^2^, median [IQR]	31 [28-36]	32 [28.5-35.5]	31 [28-36]	0.78
*Cardiovascular risk factors*, n (%)				
Hypertension	171 (73%)	38 (81%)	133 (71%)	0.18
Current or former smoker	106 (45%)	26 (55%)	80 (43%)	0.12
Dyslipidemia	159 (68%)	34 (72%)	125 (67%)	0.47
Diabetes	85 (36%)	20 (43%)	65 (35%)	0.32
Insulin-requiring diabetes	35 (15%)	10 (21%)	25 (13%)	0.14
Known CAD	126 (54%)	34 (72%)	92 (49%)	**0.004**
History of MI	100 (43%)	28 (60%)	72 (39%)	**0.01**
Medications, n (%)				
Aspirin	135 (58%)	28 (60%)	107 (57%)	0.35
Beta-blockers	144 (62%)	33 (70%)	111 (59%)	0.15
ACE inhibitors/ARB	135 (58%)	32 (68%)	103 (55%)	0.15
Diuretics	82 (35%)	27 (57%)	55 (29%)	**0.0002**
Nitroglycerine therapy (short-acting or long-acting nitrates)	24 (10%)	6 (13%)	18 (10%)	0.61
Lipid-lowering agent	154 (66%)	32 (68%)	122 (65%)	0.71

*ACE*, angiotensin-converting enzyme; *ARB*, angiotensin receptor blocker; *CAD*, coronary artery disease; *MI*, myocardial infarction

### Clinical outcomes

Over the 652 days [IQR: 559 to 751 days] of follow-up, a total of 47 participants experienced a MACE event (13 nonfatal MI, 5 cardiac deaths (1 participant had MI and then cardiac death), 10 cases of delayed revascularization, and 19 hospitalizations for congestive heart failure or de novo stable angina).

### Comparative analysis

Participants with MACE had significantly worse global stress MBF, global MFR, and regional MFC_severe_ when compared with participants without (Table 2). In contrast, global rest MBF was similar among both groups. There was a significantly higher prevalence of known CAD or history of MI in participants with MACE as compared to participants without MACE (Table 1).

**Table 2:** Myocardial perfusion imaging results

	Overall population (n = 234)	MACE (n = 47)	No MACE (n = 187)	p-value
PET pharmacological stress agent, n (%)				
Adenosine	204 (87%)	40 (85%)	164 (88%)	0.56
Hemodynamics during PET/CT, median [IQR]				
Rest-HR, bpm	70 [61-78]	71 [61-81.5]	69 [61-75.5]	0.12
Stress-HR, bpm	83 [74-95]	82 [75-99]	85 [74-94.5]	0.72
Rest-SBP, mmHg	136 ± 23	134 ± 24	136 ± 22	0.6
Stress-SBP, mmHg	120 [104-137]	118 [101-131]	120 [107.5-138]	0.07
Rest-DBP, mmHg	71 ± 12	69 ± 11	71 ± 12.5	0.3
Stress-DBP, mmHg	61 [54-70]	60 [52-63.5]	62 [55-71]	0.11
Rest-RPP > 8500 mmHg/min, n (%)	153 (65%)	29 (62%)	124 (66%)	0.60
^82^Rb quantitative imaging,				
Global rest MBF, mL/min/g, median [IQR]	0.82 [0.65-1.06]	0.72 [0.51-0.925]	0.75 [0.59-0.97]	0.34
Global stress MBF, mL/min/g, median [IQR]	1.96 [1.32-2.71]	1.5 [1.08-1.87]	2.16 [1.54-2.88]	** < 0.0001**
Global stress MBF < 1.94 mL/min/g, n (%)	113 (48%)	37 (79%)	76 (41%)	** < 0.0001**
Global MFR, median [IQR]	2.39 [1.72-3.0]	1.75 [1.395-2.47]	2.49 [1.93-3.1]	** < 0.0001**
Global MFR < 1.98, n (%)	78 (33%)	29 (62%)	49 (26%)	** < 0.0001**
MFC_severe_, % of LV, median [IQR]	0 [0-9.5]	9.3 [0-30.7]	0 [0-3.7]	** < 0.0001**
MFC_severe_ > 3.2% of LV, n (%)	78 (33%)	30 (64%)	48 (26%)	** < 0.0001**

*DBP*, diastolic blood pressure; *HR*, heart rate, *LV*, left ventricle; *MBF*, myocardial blood flow; *MFC*, myocardial flow capacity; *MFR*, myocardial flow reserve; *RPP*, rate-pressure product (HR × SBP); *SBP*, systolic blood pressure

### Optimal prognostic thresholds

Using the Youden index, we calculated the maximum potential effectiveness of global stress MBF, global MFR, and regional MFC_severe_ cutoffs for MACE prediction. For global absolute myocardial perfusion measurements, a threshold of 1.94 mL/min/g for global stress MBF achieved a specificity and sensitivity of 59% and 83%, a threshold of 1.98 for global MFR achieved a specificity and sensitivity of 73% and 64%, and a threshold of 3.2% of LV for regional MFC_severe_ achieved a specificity and sensitivity of 74% and 64%. Based on these calculated optimal thresholds, global MFR as well as regional MFC_severe_ was impaired in 78 (33%) participants, whereas global stress MBF was impaired in 113 (48%) participants (Table 2).

### Univariate and multivariate analysis

**Figure 2:**
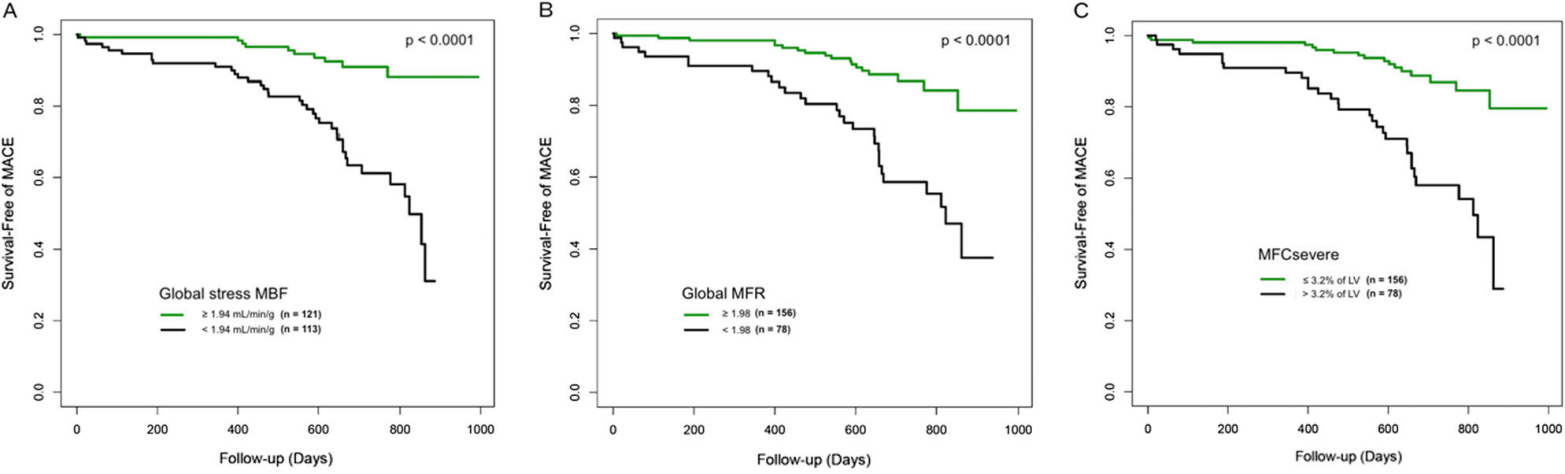
MACE-free survival curves (n = 234) according to global stress MBF (**A**), global MFR (**B**), and MFCsevere (**C**), based on optimal thresholds using the Youden Index. MACE - major adverse cardiovascular event; MBF - myocardial blood flow; MFC - myocardial flow capacity; MFR - myocardial flow reserve

The Kaplan-Meier survival curves indicated that participants with impaired global stress MBF, global MFR, or regional MFC_severe_ had significantly higher rates of MACE (all *p* < 0.0001) as compared with those with normal perfusion (Fig. 2). On univariate Cox proportional regression, global stress MBF, global MFR, and regional MFC_severe_ emerged all as significant predictors of MACE (Table 3). Male sex as well as known history of CAD and history of MI was also found to be significantly predictive of MACE (Table 3).

**Table 3:** Prediction of major adverse cardiovascular events (MACE) during follow-up. Univariate Cox proportional regression models

	MACE	
	Hazard ratio (95% CI)	p-value
Global stress MBF < 1.94 mL/min/g	5.19 (2.6-10.5)	** < 0.001**
Global MFR < 1.98	3.68 (2.0-6.6)	** < 0.001**
MFC_severe_ > 3.2% of LV	4.19 (2.3-7.6)	** < 0.001**
Age	1.02 (1.0-1.0)	0.15
Male sex	2.08 (1.1-4.1)	**0.034**
BMI	0.99 (0.9-1.0)	0.68
Hypertension	1.77 (0.9-3.7)	0.13
Current or former smoker	1.64 (0.9-2.9)	0.093
Dyslipidemia	1.41 (0.7-2.7)	0.3
Diabetes	1.52 (0.9-2.7)	0.17
Known CAD	2.41 (1.3-4.6)	**0.005**
History of MI	2.19 (1.2-3.9)	**0.008**

BMI - body mass index; other abbreviations as in Tables 1 and 2

In multivariate analysis with 3 separate models including each time clinical risk factors and separately global stress MBF, global MFR, and regional MFC_severe_, each one of these PET variables emerged as powerful independent predictors of MACE (Table 4). In contrast, clinical variables such as male sex, known CAD, and history of MI did not (Table 4).

**Table 4:** Independent predictors of MACE in different Cox regression models

Multivariate model	Hazard ratio (95% CI)	p-value
*Model 1: Clinical variables and global stress MBF*		
Global stress MBF < 1.94 mL/min/g	4.5 (2.1-9.7)	** < 0.001**
Male sex	1.03 (0.5-2.2)	0.94
Known CAD	1.42 (0.5-3.7)	0.47
History of MI	1.03 (0.4-2.5)	0.94
*Model 2: Clinical variables and global MFR*		
Global MFR < 1.98	3.1 (1.7-5.8)	** < 0.001**
Male sex	1.33 (0.7-2.8)	0.47
Known CAD	1.76 (0.7-4.5)	0.24
History of MI	0.96 (0.4-2.3)	0.93
*Model 3: Clinical variables and MFC*_*severe*_		
MFC_severe_ > 3.2% of LV	3.67 (1.9-7.1)	** < 0.001**
Male sex	1.48 (0.7-3.0)	0.29
Known CAD	1.58 (0.6-4.1)	0.35
History of MI	0.81 (0.3-2.0)	0.64
*Model 4: Clinical variables, global MFR, global stress MBF, and MFC*_*severe*_		
Global stress MBF < 1.94 mL/min/g	2.81 (1.1-7)	**0.027**
Global MFR < 1.98	1.54 (0.7-3.7)	0.33
MFC_severe_ > 3.2% of LV	1.56 (0.6-4.1)	0.37
Male sex	1.05 (0.5-2.3)	0.9
Known CAD	1.59 (0.6-4.2)	0.35
History of MI	0.82 (0.3-2)	0.67

Abbreviations as in Tables 1 and 2

Moreover, for the most comprehensive model including clinical parameters and all global MFR, global stress MBF, and regional MFC_severe_, only global stress MBF emerged as an independent prognostic factor for MACE (hazard ratio (HR) 2.81, *p* = 0.027), while global MFR and regional MFC_severe_ did not (Table 4).

## Discussion

Our results support that impaired global stress MBF, global MFR, and regional MFC_severe_, as assessed by using low-dose ^82^Rb with the latest PET SiPM technology, allowing halving of the standard injected activity and radiation dose, are powerful predictors of cardiovascular events, outperforming traditional cardiovascular risk factors such as the presence of known CAD or history of MI. In a comprehensive analysis, we found that only reduced global stress MBF independently predicted MACE.

PET myocardial perfusion imaging is well established for the diagnostic and prognostic evaluation of patients with suspected CAD. Novel PET cameras using SiPM detectors offer a considerable advantage in radiation dose compared with the conventional PET cameras. For a 70-kg patient, the effective radiation dose could be reduced from > 4-mSv with the conventional PET cameras to < 1-mSv.^18^ However, no previous clinical study has evaluated the prognostic value of PET myocardial perfusion imaging with SiPM.

The independent prognostic value of reduced global stress MBF, being superior to global MFR and regional MFC_severe_ impairments, was unexpected and is discrepant as compared to previous studies. Regarding the prediction of cardiovascular deaths, Gupta et al. investigated the importance of global stress MBF and MFR.^10^ In this study, in multivariate analysis, the authors reported that the cardiovascular mortality was independently driven by global MFR, irrespective of whether the global stress MBF was impaired or preserved. Similar findings had been reported in the retrospective study by Fukushima et al.^19^ with a similar sample size as compared to the current study (n = 224 vs. n = 234 in the current study). However, compared with Fukushima et al.^19^ study, the current study has a longer follow-up (median, 652 vs. 426 days), and population as well as medication use depicted some difference (male sex, 65% vs. 40%; prior history of MI, 43% vs. 11%; beta-blockers, 62% vs. 18%). Taqueti et al. demonstrated that the prognostic value of MFR for the occurrence of MACE was independent of the extent and severity of coronary lesions as evaluated on coronary angiography, but stress MBF was not included in multivariate analysis.^20^ In an observational study by Patel et al., a threshold of 1.8 for global MFR has been identified to yield a benefit of coronary revascularization over medical treatment, independently of means of revascularization or the extent of myocardial ischemia on semi-quantitative analysis, but, again, stress MBF was not studied.^21^

Similar to the current cohort, other studies reported that global stress MBF was independently predictive for events, whereas global MFR was not. Global stress MBF was shown to be superior to global MFR for the prediction of MACE in a ^82^Rb PET/CT study by Farhad et al.^18^, and for the prediction of a composite of death and MI in a recent [^15^O]H_2_O PET/CT study by Bom et al.^22^. Since MFR is inherently dependent on resting flow, which is known to be highly sensitive to hemodynamic conditions, impairment of MFR may be less specific for the occurrence of events. However, importantly, both the study by Bom et al. and the current cohort used adjusted MFR for the resting rate-pressure product, accounting at least partially for changes in resting flow caused by differences in hemodynamic conditions. The present finding of a superiority of global stress MBF vs. global MFR impairments is consistent with the lack of association between resting MBF and clinical prognosis.^23^

Studies evaluating the prognostic value of MFC in comparison with stress MBF and MFR are scarce. Gould et al. showed in two recent observational studies with large cohorts over long-term follow-up that the extent of severe regional impairment of MFC, expressed as percent of LV as in the current cohort, provides optimal risk stratification and is associated with a survival benefit gain after revascularization.^8,9^ This risk stratification after revascularization was better assessed by severe regional MFC alteration rather than global MBF alteration.^9^ Recent studies using modified MFC with average MFR and stress MBF per coronary territory have shown an association with cardiovascular death and MACE.^24,25^

Considering other regional quantitative measures, our results are consistent with Bom et al. who found in a [^15^O]H_2_O PET/CT study that both global and regional stress MBF have prognostic value in predicting cardiac events, while the combination of global and regional stress MBF did not improve the prognostic performance compared to either alone.^22^ Harjulahti et al. found similar results using also [^15^O]H_2_O PET/CT.^4^ In contrast, Von Felten et al. recently demonstrated using ^13^ N-ammonia, an independent prognostic value of regional MFR < 2, being superior to global stress MBF and MFR.^26^ In the current study, the first using the latest SiPM technology, whose characteristics might offer potential benefits for precise analysis, we found that severe regional per pixel MFC impairment is a powerful predictor of cardiovascular events, independent from traditional cardiovascular risk factors, but not from global perfusion parameters. Although the current study has inherent limitations which could limit the scope of the results, these data support a close relationship between global and regional perfusion.^22^

### Limitations

This study must be interpreted in the context of its single-center design, with an average sample size despite a high completeness of follow-up, which still limits extensive subgroup analysis. The follow-up period was middle range, with a low incidence of hard cardiac events such as cardiac death. The present thresholds for the MFC_severe_ category (pixel having both MFR ≤ 1.5 and stress MBF ≤ 1.1 mL/min/g) were slightly different as compared to the thresholds used by Johnson and Gould (pixel having both MFR ≤ 1.27 and stress MBF ≤ 0.83 mL/min/g). However, significant contributions of these slight differences to the present results seem unlikely. Furthermore, currently available SiPM cameras differ significantly in design, and it cannot be excluded that different SiPM cameras have different prognostic ability. The current study emphasized on myocardial blood flow measurements, and LV ejection fraction, LV volumes, regional wall motion, coronary artery calcium score, and semi-quantitative evaluation of relative perfusion defects were not assessed in this study based on myocardial blood flow quantification, despite representing important information that could be part of routine PET/CT imaging. Finally, the current prognostic study did not assess diagnostic accuracy, which could be a strength of the quantification of regional perfusion with MFC.

## New Knowledge Gained

This study shows the prognostic value of impaired stress MBF, MFR, and MFC for cardiovascular event using low-dose ^82^Rb SiPM PET/CT technology with halved injected activity delivering < 1.0-mSv radiation dose for a 70-kg patient.

## Conclusion

In conclusion, this study using the latest SiPM PET technology with low-dose ^82^Rb, halving the standard activity, demonstrates that impaired global stress MBF, global MFR, and regional MFC are powerful predictors of cardiovascular events, outperforming traditional cardiovascular risk factors. However, we found that only reduced global stress MBF independently predicted MACE, being superior to global MFR and regional MFC impairments.

## Supplementary Information

Below is the link to the electronic supplementary material.

Supplementary file1 (PPTX 2762 KB)

Supplementary file2 (DOCX 725 KB)

Supplementary file3 (M4A 7591 KB)

## Abbreviations

MBF: Myocardial blood flow
MFR: Myocardial flow reserve
PET: Positron emission tomography
CAD: Coronary artery disease
MFC: Myocardial flow capacity
SiPM: Silicon photomultipliers with digital readout
^82^Rb: Rubidium-82
LV: Left ventricle
MACE: Major adverse cardiovascular event
MI: Myocardial infarction
