# Supplementary material for: Comparison of the prognostic value of impaired stress myocardial blood flow, myocardial flow reserve, and myocardial flow capacity on low-dose Rubidium-82 SiPM PET/CT
Source: J Nucl Cardiol. 2022 Dec 27;30(4):1385–95. doi: 10.1007/s12350-022-03155-6 (PMC10371877; doi:10.1007/s12350-022-03155-6)
Supplement: Supplementary file 1 — Supplementary file1 (PPTX 2762 KB) [file 12350_2022_3155_MOESM1_ESM.pptx]

## Slide 1
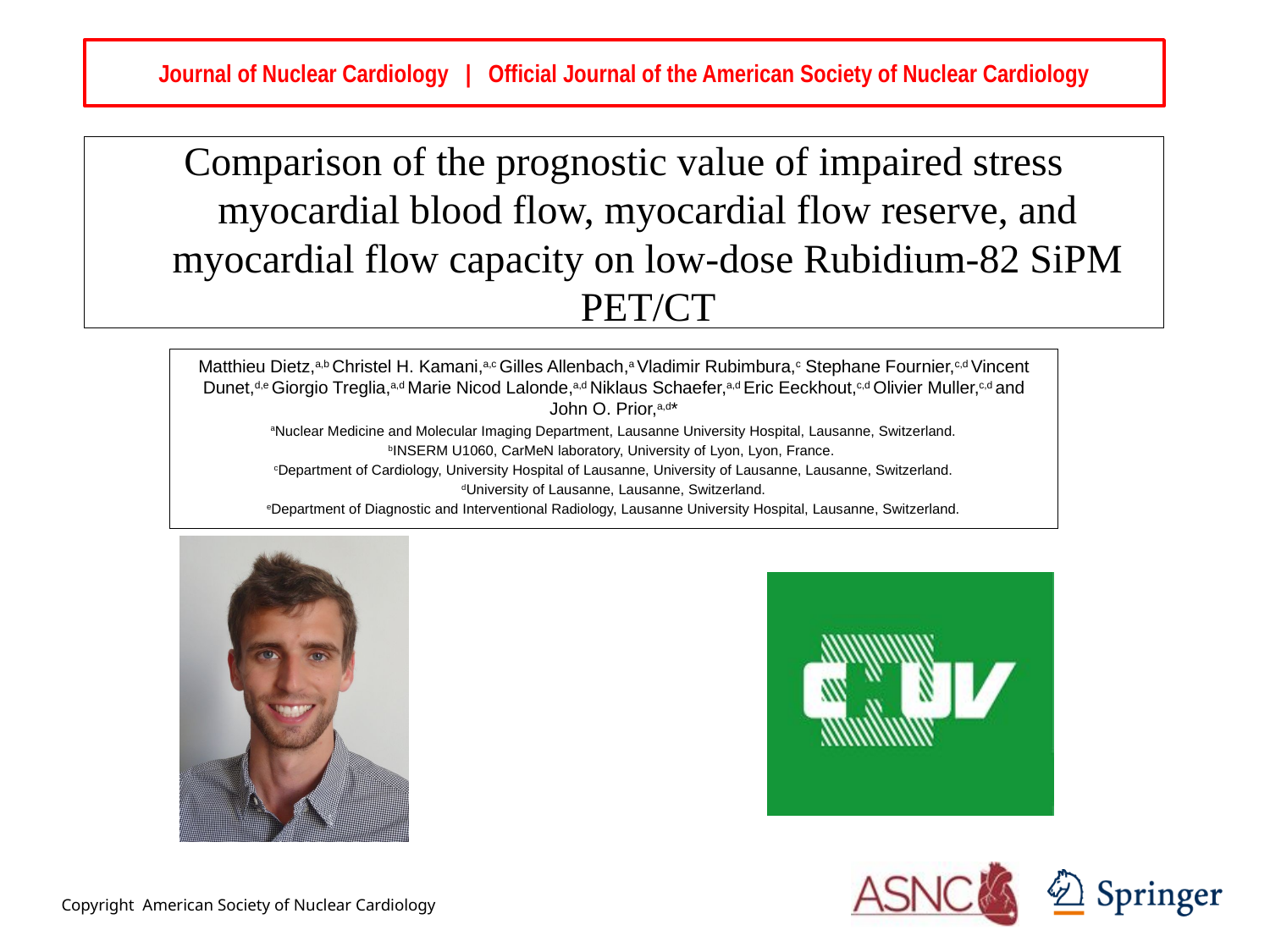

Journal of Nuclear Cardiology | Official Journal of the American Society of Nuclear Cardiology
# Comparison of the prognostic value of impaired stress myocardial blood flow, myocardial flow reserve, and myocardial flow capacity on low-dose Rubidium-82 SiPM PET/CT
Matthieu Dietz,a,b Christel H. Kamani,a,c Gilles Allenbach,a Vladimir Rubimbura,c Stephane Fournier,c,d Vincent Dunet,d,e Giorgio Treglia,a,d Marie Nicod Lalonde,a,d Niklaus Schaefer,a,d Eric Eeckhout,c,d Olivier Muller,c,d and John O. Prior,a,d*
aNuclear Medicine and Molecular Imaging Department, Lausanne University Hospital, Lausanne, Switzerland.
bINSERM U1060, CarMeN laboratory, University of Lyon, Lyon, France.
cDepartment of Cardiology, University Hospital of Lausanne, University of Lausanne, Lausanne, Switzerland.
dUniversity of Lausanne, Lausanne, Switzerland.
eDepartment of Diagnostic and Interventional Radiology, Lausanne University Hospital, Lausanne, Switzerland.
Copyright American Society of Nuclear Cardiology

## Slide 2
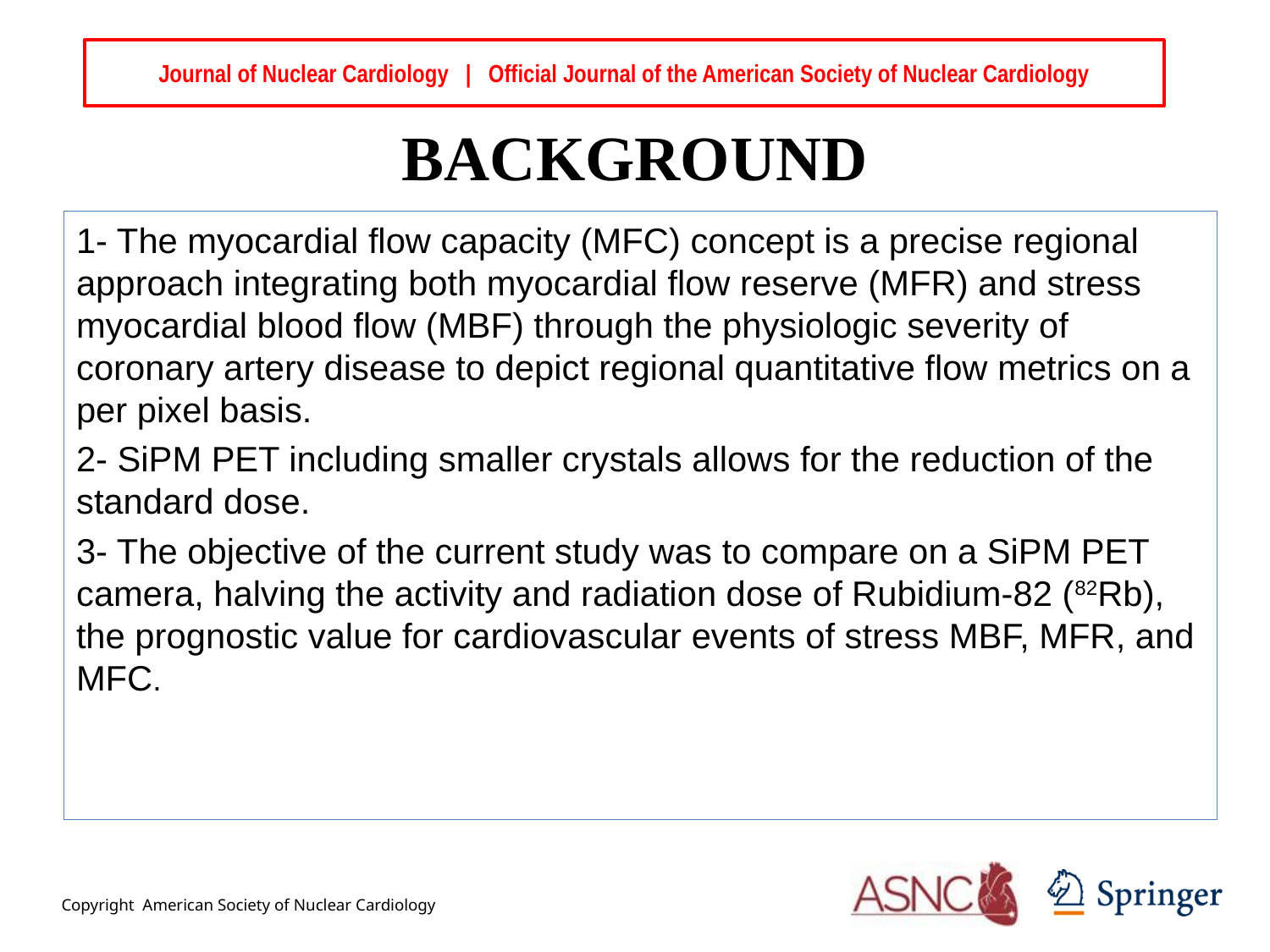

Journal of Nuclear Cardiology | Official Journal of the American Society of Nuclear Cardiology
# BACKGROUND
1- The myocardial flow capacity (MFC) concept is a precise regional approach integrating both myocardial flow reserve (MFR) and stress myocardial blood flow (MBF) through the physiologic severity of coronary artery disease to depict regional quantitative flow metrics on a per pixel basis.
2- SiPM PET including smaller crystals allows for the reduction of the standard dose.
3- The objective of the current study was to compare on a SiPM PET camera, halving the activity and radiation dose of Rubidium-82 (82Rb), the prognostic value for cardiovascular events of stress MBF, MFR, and MFC.
Copyright American Society of Nuclear Cardiology

## Slide 3
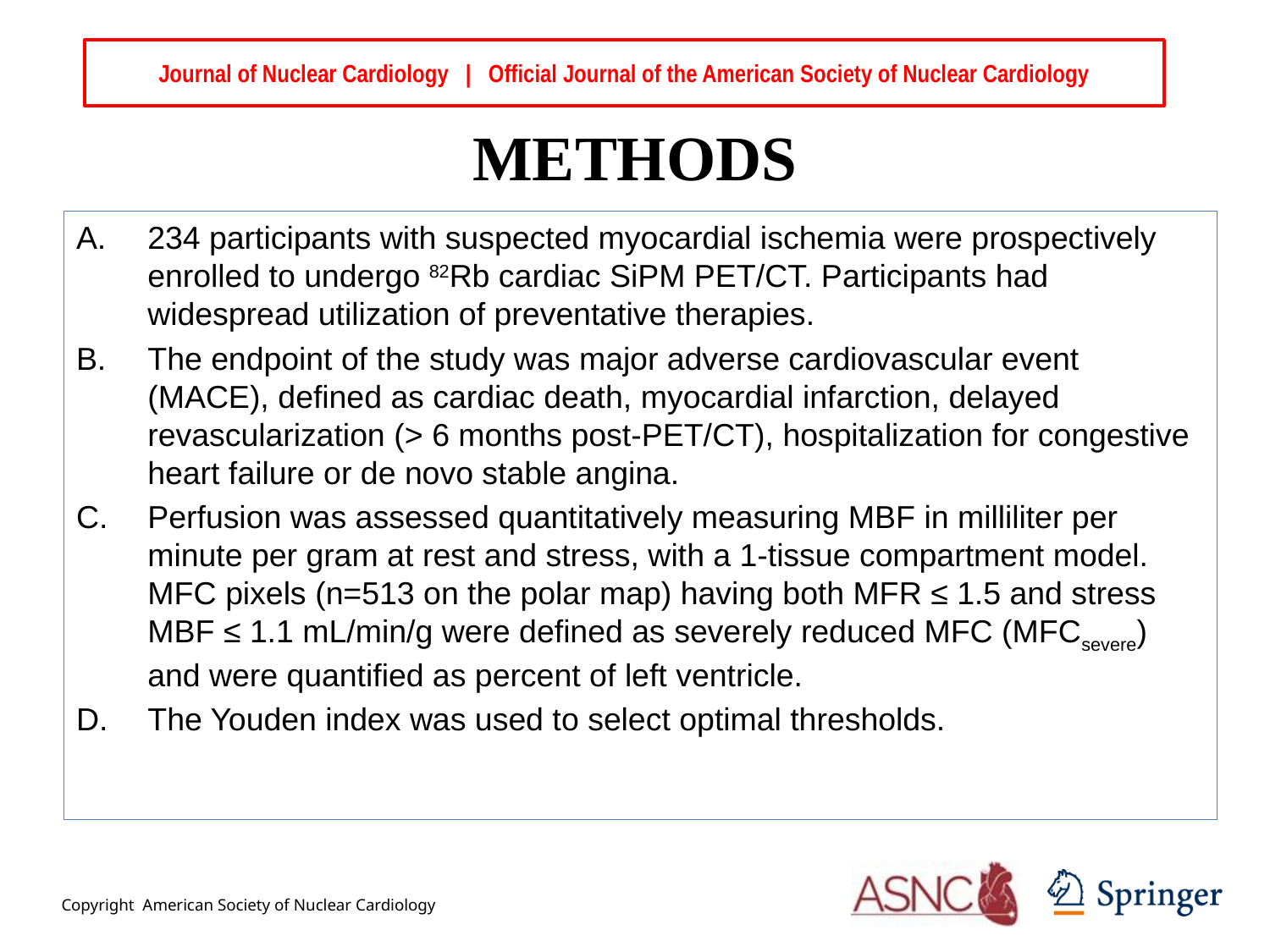

Journal of Nuclear Cardiology | Official Journal of the American Society of Nuclear Cardiology
# METHODS
234 participants with suspected myocardial ischemia were prospectively enrolled to undergo 82Rb cardiac SiPM PET/CT. Participants had widespread utilization of preventative therapies.
The endpoint of the study was major adverse cardiovascular event (MACE), defined as cardiac death, myocardial infarction, delayed revascularization (> 6 months post-PET/CT), hospitalization for congestive heart failure or de novo stable angina.
Perfusion was assessed quantitatively measuring MBF in milliliter per minute per gram at rest and stress, with a 1-tissue compartment model. MFC pixels (n=513 on the polar map) having both MFR ≤ 1.5 and stress MBF ≤ 1.1 mL/min/g were defined as severely reduced MFC (MFCsevere) and were quantified as percent of left ventricle.
The Youden index was used to select optimal thresholds.
Copyright American Society of Nuclear Cardiology

## Slide 4
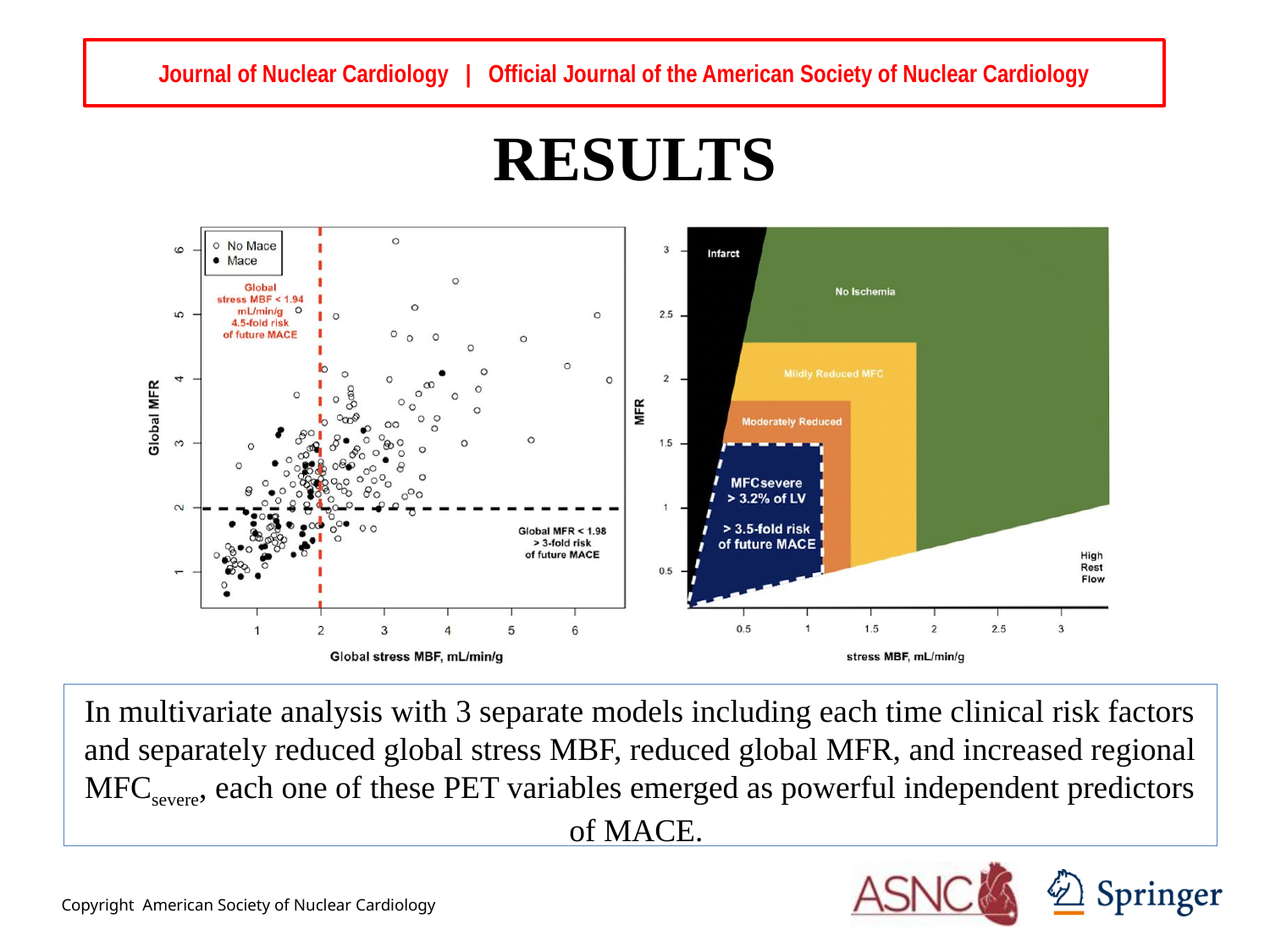

Journal of Nuclear Cardiology | Official Journal of the American Society of Nuclear Cardiology
# RESULTS
In multivariate analysis with 3 separate models including each time clinical risk factors and separately reduced global stress MBF, reduced global MFR, and increased regional MFCsevere, each one of these PET variables emerged as powerful independent predictors of MACE.
Copyright American Society of Nuclear Cardiology

## Slide 5
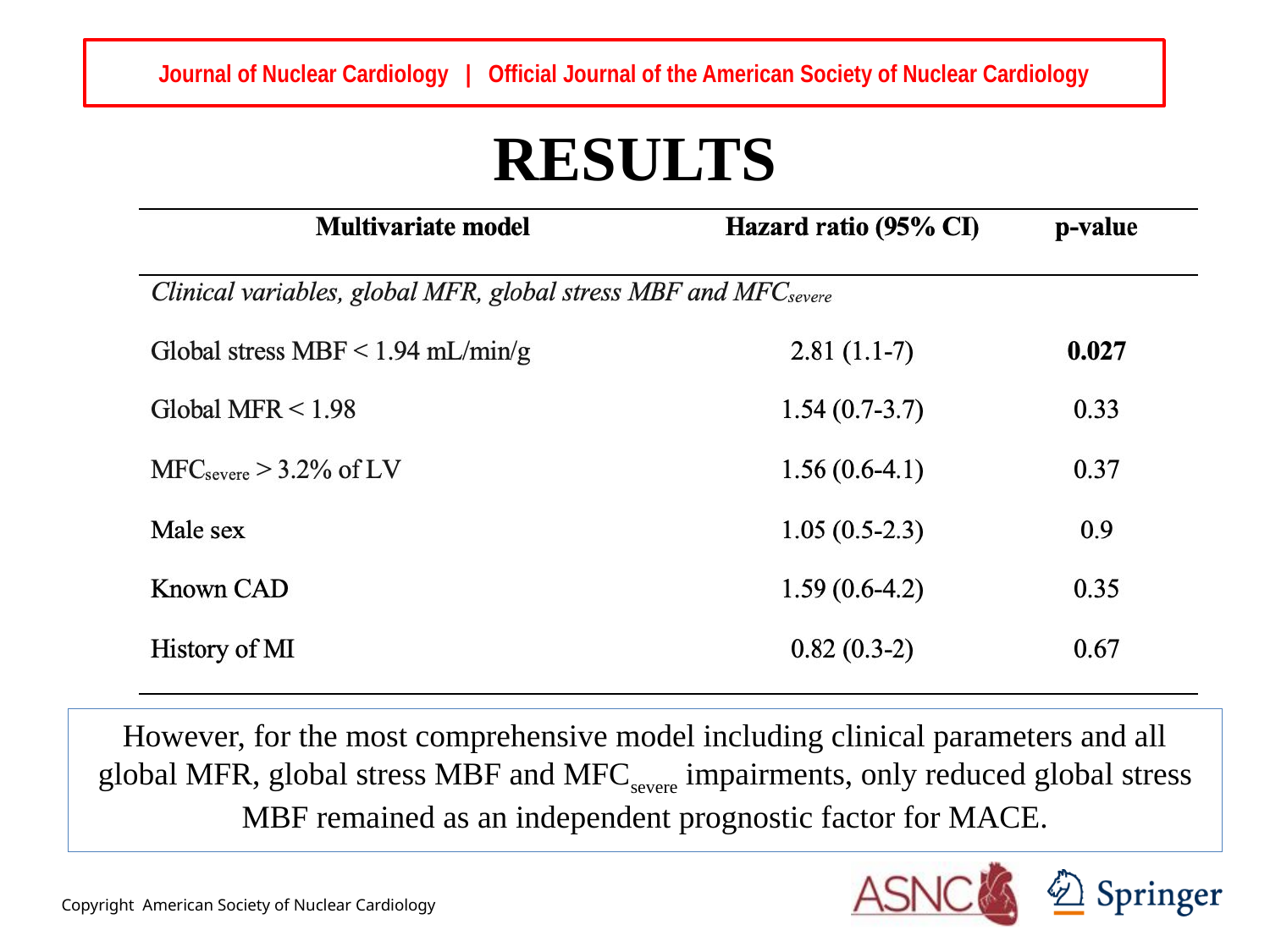

Journal of Nuclear Cardiology | Official Journal of the American Society of Nuclear Cardiology
# RESULTS
However, for the most comprehensive model including clinical parameters and all global MFR, global stress MBF and MFCsevere impairments, only reduced global stress MBF remained as an independent prognostic factor for MACE.
Copyright American Society of Nuclear Cardiology

## Slide 6
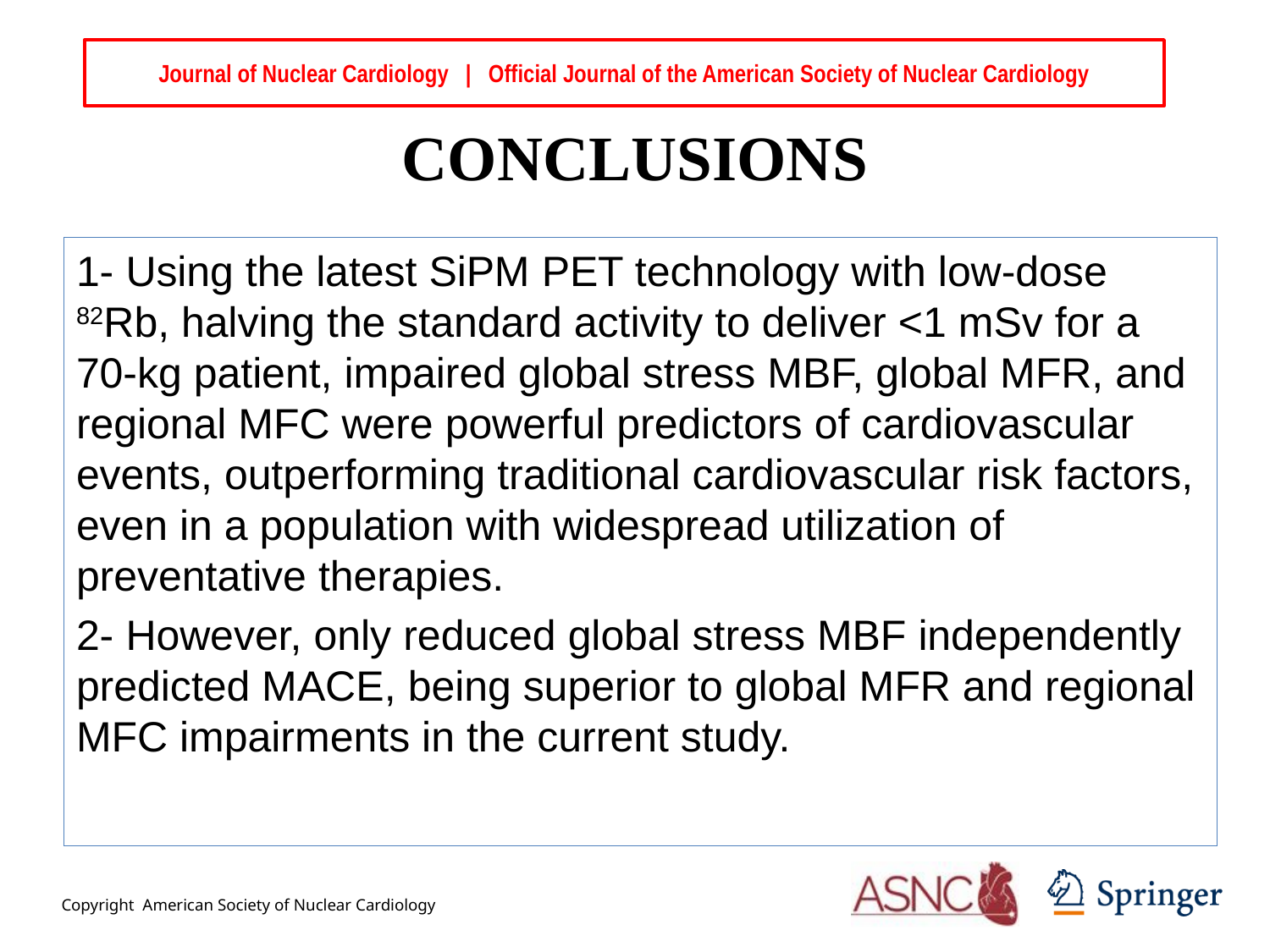

Journal of Nuclear Cardiology | Official Journal of the American Society of Nuclear Cardiology
# CONCLUSIONS
1- Using the latest SiPM PET technology with low-dose 82Rb, halving the standard activity to deliver <1 mSv for a 70-kg patient, impaired global stress MBF, global MFR, and regional MFC were powerful predictors of cardiovascular events, outperforming traditional cardiovascular risk factors, even in a population with widespread utilization of preventative therapies.
2- However, only reduced global stress MBF independently predicted MACE, being superior to global MFR and regional MFC impairments in the current study.
Copyright American Society of Nuclear Cardiology
