# Supplementary material for: Comparison of the prognostic value of impaired stress myocardial blood flow, myocardial flow reserve, and myocardial flow capacity on low-dose Rubidium-82 SiPM PET/CT
Source: J Nucl Cardiol. 2022 Dec 27;30(4):1385–95. doi: 10.1007/s12350-022-03155-6 (PMC10371877; doi:10.1007/s12350-022-03155-6)

#JournalNC: Using SiPM #PET with low-dose ^82^Rb, delivering <1 mSv for a 70-kg patient, impaired global stress MBF, global MFR, and regional MFC are powerful predictors of #MACE, even despite widespread utilization of preventative therapies!

@chuvlausanne @MyASNC @CVPET


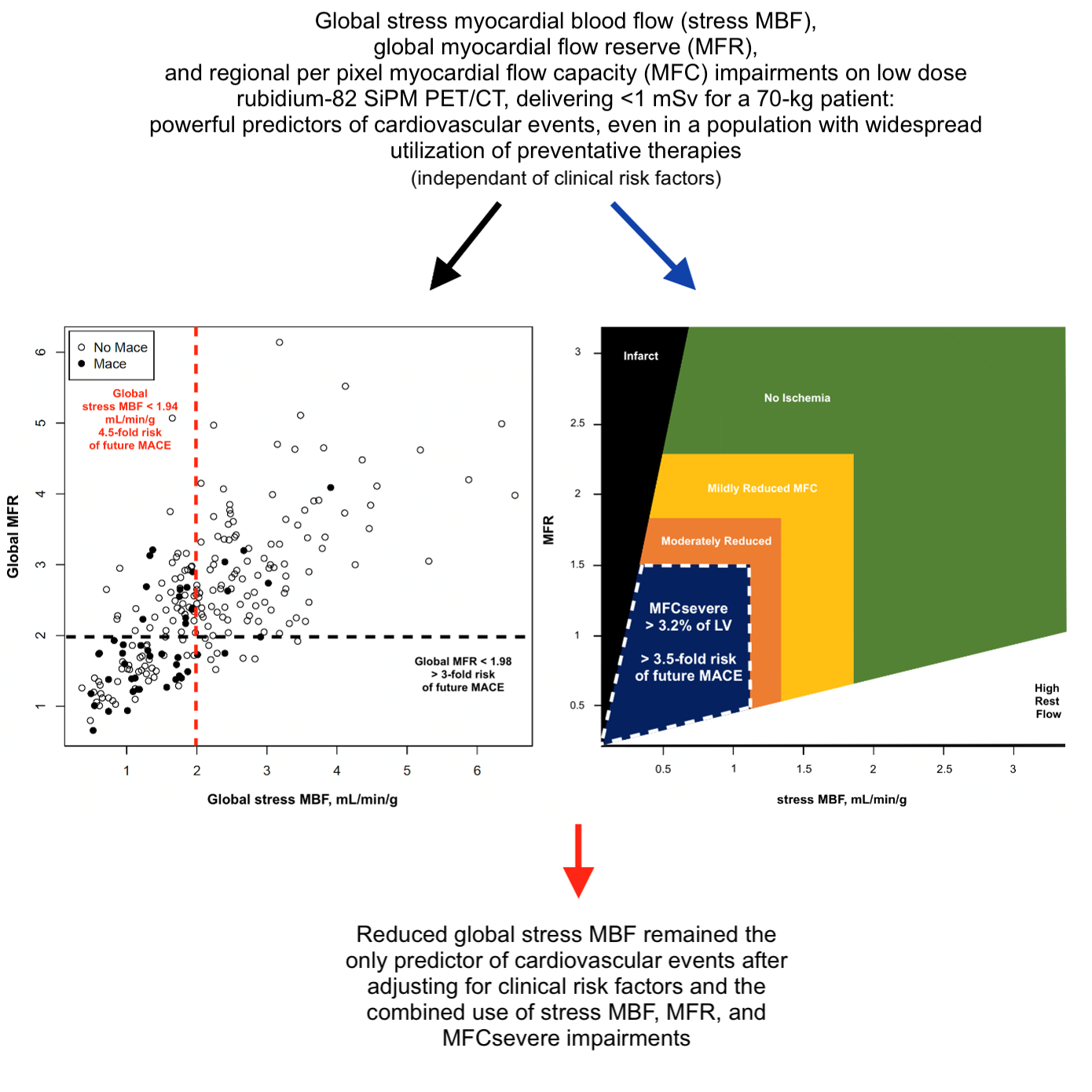

Supplement: Supplementary file 2 — Supplementary file2 (DOCX 725 KB) [file 12350_2022_3155_MOESM2_ESM.docx]
